# Genetic abrogation of the fibronectin-α5β1 integrin interaction in articular cartilage aggravates osteoarthritis in mice

**DOI:** 10.1371/journal.pone.0198559

**Published:** 2018-06-05

**Authors:** Maylin Almonte-Becerril, Irene Gimeno-LLuch, Olga Villarroya, María Benito-Jardón, Juan Bautista Kouri, Mercedes Costell

**Affiliations:** 1 Department of Biochemistry and Molecular Biology and Estructura de Reserca Interdisciplinar en Biotecnologia i Biomedicina, Universitat de València, Burjassot, Spain; 2 Departamento de Infectómica y Patogénesis Molecular, Centro de investigación y de Estudios Avanzados del Instituto Politécnico Nacional (CINVESTAV-IPN), México D.F., México; University of Bergen, NORWAY

## Abstract

The balance between synthesis and degradation of the cartilage extracellular matrix is severely altered in osteoarthritis, where degradation predominates. One reason for this imbalance is believed to be due to the ligation of the α5β1 integrin, the classic fibronectin (FN) receptor, with soluble FN fragments instead of insoluble FN fibrils, which induces matrix metalloproteinase (MMP) expression. Our objective was to determine whether the lack of α5β1-FN binding influences cartilage morphogenesis *in vivo* and whether non-ligated α5β1 protects or aggravates the course of osteoarthritis in mice. We engineered mice (*Col2a-Cre;Fn1*^*RGE/fl*^), whose chondrocytes express an α5β1 binding-deficient FN, by substituting the aspartic acid of the RGD cell-binding motif with a glutamic acid (FN-RGE). At an age of 5 months the knee joints were stressed either by forced exercise (moderate mechanical load) or by partially resecting the meniscus followed by forced exercise (high mechanical load). Sections of femoral articular knees were analysed by Safranin-O staining and by immunofluorescence to determine tissue morphology, extracellular matrix proteins and matrix metalloproteinase expression. The articular cartilage from untrained control and *Col2a-Cre;Fn1*^*RGE/fl*^ mice was normal, while the exposure to high mechanical load induced osteoarthritis characterized by proteoglycan and collagen type II loss. In the *Col2a-Cre;Fn1*^*RGE/fl*^ articular cartilage osteoarthritis progressed significantly faster than in wild type mice. Mechanistically, we observed increased expression of MMP-13 and MMP-3 metalloproteinases in FN-RGE expressing articular cartilage, which severely affected matrix remodelling. Our results underscore the critical role of FN-α5β1 adhesion as ECM sensor in circumstances of articular cartilage regeneration.

## Introduction

The normal net balance between synthesis and degradation of cartilage extracellular matrix (ECM) is severely altered in osteoarthritis (OA), where degradation predominates and eventually results in the destruction of the articular cartilage. Currently, enormous efforts are dedicated to design therapies that revert or at least stop cartilage destruction. Tensions on the articular cartilage such as high impact exercises produce injuries that initiate a process of regeneration with limited potential due to the avascular nature of cartilage [1]. Another adverse factor for efficient cartilage ECM remodelling during regeneration is the formation of fragments from ECM components such as type II collagen, fibronectin (FN) and aggrecan, that, if they persist, promote the expression of cytokines and matrix metalloproteinases (MMPs). For example, FN fragments, but not full length FN molecules were shown to contribute to disease progression [2–4] by accelerating MMPs production [5] and up-regulating the expression of catabolic cytokines in chondrocytes [6,7].

FN is a matrix glycoprotein composed of an array of type I, II and III modules. FN forms fibrillar matrices by an integrin-mediated assembly process. FN also binds collagens and heparan sulphate proteoglycans and offers multiple binding sites to cell adhesion molecules such as integrins and syndecans [8]. Since chondrocytes lack or have very few cell-to-cell contacts, it is thought that cell-matrix adhesions are major regulators of cell proliferation and differentiation [9,10]. FN is not essential for cartilage development, as mice with a cartilage-specific deletion of the FN gene lack obvious alterations in bone and cartilage [11]. However, the FN function during cartilage injury and regeneration has not been studied, although its potential role for cartilage regeneration is controversially discussed. While some authors found FN to promote ECM remodelling and chondrocyte survival and proliferation [12,13], others observed tissue-damaging pro-inflammatory effects induced by FN fragments [14–17].

The major cell-binding region of FN is the RGD motif, located in the FNIII10 repeat. The RGD motif binds α5β1 and αv-class integrins and controls FN fibrils assembly [8]. The α5β1 and αvβ5 integrins are the major integrins responsible for chondrocyte adhesion to FN [18]. It was also shown that α5β1 is heavily up-regulated in the OA cartilage [19]. FN fragments including the FNIII10 region have received particular attention as they bind α5β1 integrins on chondrocytes leading to rapid elevation of intracellular levels of reactive oxygen species and the production of MMP-13 that in turn further accelerates cartilage degradation [14–17]. Moreover, inhibition of α5 integrin expression was shown to arrest cartilage destruction induced by FN fragments in *in vitro* explant experiments [16]. These data, together with reports showing that the inhibition of MMP-13 expression or activation protects from inflammation, joint destruction [20–22] and experimental OA progression [23], underline the importance of understanding the α5β1 integrin adhesion and signalling and identify α5β1 as therapeutic target to halt OA progression.

The purpose of this study was to directly test whether the *in vivo* suppression of FN-RGD-mediated adhesion to α5β1 integrins in a genetically engineered mouse model influences cartilage morphogenesis and if not, whether the FN-RGD site alters both cartilage stability and the course of OA after experimental induction. We used a transgenic mouse strain in which the RGD motif in the FN gene was converted into RGE leading to a failure of α5β1 integrins to bind FN without affecting FN binding to αv-class integrins [24,25]. To express exclusively FN-RGE in cartilage we disrupted the floxed FN gene in compound heterozygous animals (*Fn1*^*RGE/fl*^) using a collagen type II-driven Cre transgenic strain [26]. We demonstrate that mice lacking α5β1-FN binding in cartilage (*Fn1*^*RGE/-*^) develop normal cartilage and bone. After moderate mechanical load, induced by forced exercise for 10 days, the knee cartilage remained unaffected although matrix metalloproteinase MMP-3 and MMP-13 levels were elevated. However, after exposure to high mechanical load, induced by partial meniscus resection followed by forced exercise, the OA progressed much faster in mice expressing FN-RGE in cartilage and was accompanied by elevated matrix metalloproteinase expression and by a dramatic reduction of cartilage ECM.

## Materials and methods

### Mice

The mice were housed in special pathogen free animal facility of the University of Valencia, Spain. We used the *Fn1*^*RGE*^ mouse strain in which the aspartic acid (D) of the RGD motif of FN was substituted with a glutamic acid (E) [24,25]. Since *Fn1*^*RGE/RGE*^ mice die at E9.5, we intercrossed *Fn1*^*+/RGE*^, *Fn1*^*+/fl*^ [26] and murine type II collagen promotor-driven (*Col2a1*)-Cre [27] mouse strains to obtain the *Col2a1-cre;Fn1*^*RGE/fl*^ line (*Fn1*^*RGE/-*^) that lacks the floxed FN allele and hence only expresses FN-RGE in cartilage (S1 Fig). It was previously demonstrated that the FN expression in cartilage is efficiently abrogated with *Col2a1-cre;Fn1*^*fl/fl*^ mice [11]. Littermate *Col2a1-cre;Fn1*^*wt/wt*^ (*Fn1*^*wt/wt*^) mice were used as control group. Genotyping was carried out by PCR in DNA derived from tail (T) biopsies and the Cre-mediated deletion of the floxed FN allele was confirmed in DNA from the femoral capsules (C). The PCR primers used to identify the different alleles were as follows: for *Fn1*^RGE^ 5’-CAAAGAAGACCCCAAGAGCA-3’ (forward) and 5’-ACAAGCCCTGGCCTTTAGTT-3’ (reverse); for *Fn1*^*fl*^ 5’-GTACTGTCCCATATAAGCCTCTG-3’ (forward) and 5’-CTGAGCATCTTGAGTGGATGG GA-3’ (reverse). The location of the primers in the *Fn1* gene is depicted in S1 Fig. The Cre transgene was identified with: 5’-GCCTGCATTACCGGTCGATGCAACGA-3’ (forward) and 5’-GTGGCAGATGGCGCGGCAACACCATT-3’ (reverse).

### OA induction and forced exercise (FEx)

OA was induced in 5-month-old male *Fn1*^*RGE/-*^ and *Fn1*^*wt/wt*^ mice by surgically resecting the medial meniscus in the back right leg knee joint and exposing the mice to forced exercise, which consisted of walking daily a distance of 900 cm on a 0.5 cm wide bar followed by 10 jumps from a 10 cm height. This exercise, called here high load, was shown to accelerate the apparition of OA in rats after partial menisectomy [19,28]. In the case of our wild type mouse strain, this procedure induced progressive cartilage degeneration in 15–20 days. To expose *Fn1*^*RGE/-*^ articular cartilage to a milder stress, we used the same protocol of forced exercise, however without menisectomy. This protocol results in moderate mechanical load and is called forced exercise (FEx). Both procedures were approved by the Animal Care and Ethics Committee of the Government of the Valencian Community (permission reference A1337246081246).

### Histological analyses and tissue stainings

At indicated time points mice were sacrificed by cervical dislocation, and the whole knee joints dissected and fixed in 4% paraformaldehyde (PFA) buffered with phosphate buffer saline (PBS) for 24 h at 4°C. The joints were decalcified for 18 h with 1:1 (v:v) 20% formic acid and 10% sodium citrate at 4°C. After dehydration with an increasing concentration series of ethanol (70%, 80%, 90%, 100%) and embedding in Paraplast (Sigma-Aldrich), 6 μm sagittal sections were prepared from the medial compartment of the joints, stained with Safranin-O or processed for immunostaining.

For Safranin-O staining sections were hydrated with PBS, stained sequentially with 0.01% fast green (Cymit, Barcelona), 1% acetic acid (Sigma-Aldrich) and 1% Safranin-O, washed in running tap water, dehydrated by crescent concentrations of ethanol (90%, 50%, 70%, 90%, 96%, and 100%), incubated in xylol, and finally mounted with entellan mounted medium (Merck, Germany).

For Haematoxylin-Chromotrope-2R staining, sections were hydrated, stained for 4 min with Mayer’s hematoxylin solution (Sigma-Aldrich), washed and stained for 4 min in 1% Chromotrope (Santa Cruz) in ethanol 95%.

For immunostaining sections were re-hydrated with decreasing concentrations of ethanol (100%, 95%, 80%, 70%) and PBS. Sections were pretreated with 2 mg/ml hyaluronidase (Sigma Chemical, Germany) in PBS for 30 min at 37°C. Afterwards, sections were permeabilized with 0.2% tween-20 in PBS, pre-incubated with 0.2% IgG-free bovine serum albumin (Sigma-Aldrich) for 20 min at room temperature and incubated overnight at 4°C with primary antibodies.

The following primary antibodies were used: mouse anti-type II collagen (1:200, Santa Cruz Biotech), goat anti-type I collagen (1:200, Santa Cruz Biotechnology), rabbit anti-MMP-3 (1:200, Abcam), rabbit anti-MMP-13 (1:200, Abcam); rabbit anti-FN (1:250, Millipore) and rabbit anti-aggrecan (1:50, Millipore).

Secondary anti-mouse, -goat or -rabbit antibodies labeled with Cy3 (red) were used for immunofluorescence, and secondary biotinylated anti-IgG (1:200; Vector Laboratories) antibody were used for immunohistochemistry assays. Nuclei were stained with DAPI (1:600; Invitrogen, Paisley UK) for 1 min. The sections were coverslipped in Vectashield mounting medium (Vector Laboratories, Burlingame, CA, USA). Immunostaining was visualized with ABC Elite kit (Vector Laboratories) and a solution of diaminobenzidine (Sigma-Aldrich) and hydrogen peroxide.

For negative controls the primary antibody was omitted. To quantify the fluorescence in cartilage the stained sections were observed with a Leica confocal microscope (TCS-SP5-DMI6000B with Plan Neor Fluor) and the number of pixels in the cartilage zone was evaluated and referred to the area size, using the Leica LAS AF lite confocal program. In each mouse, 3 sections were analyzed and in each section 3 randomly picked different fields were quantified. For statistical analysis we used the Tukey–Kramer multiple comparison test.

### OA grading

The cartilage alterations were scored using the OARSI histopathology recommendations [29]. This semi-quantitative scoring system was applied to 10 images of the femoral condyles, separately scored by four researchers. The linear scale is applied to the medial and lateral quadrants of the joint. The OA severity is expressed as maximal scores that can be observed in the entire joint. Grade 0 is normal cartilage.

## Results

### FN-RGE enables normal bone and cartilage development

**Fig 1:**
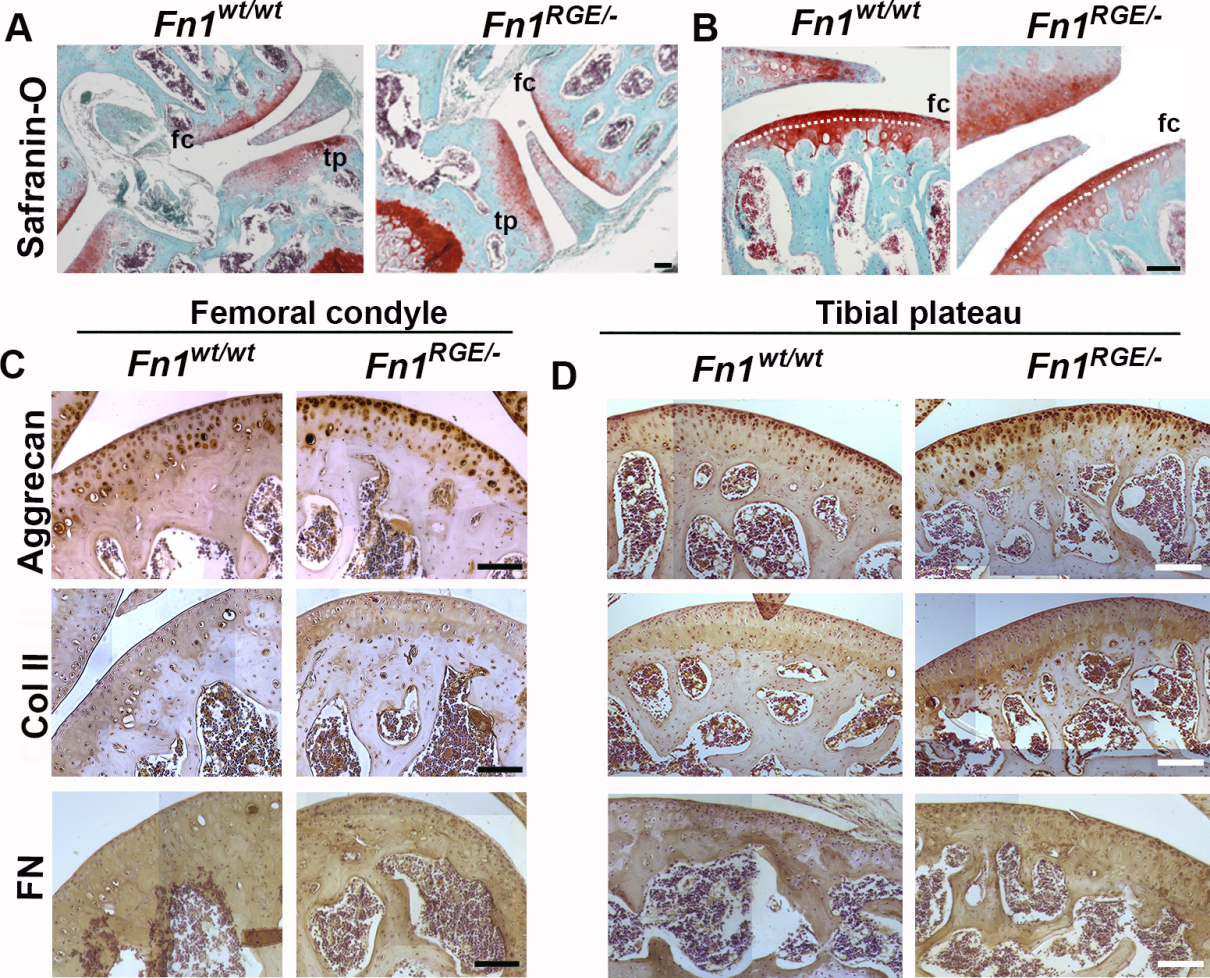
Morphology of articular cartilage in *Fn1*^*RGE/-*^ mice. A) Representative Safranin-O stained sections from wild type (*Fn1*^*wt/wt*^) and mutant (*Fn1*^*RGE/-*^) knee joint cartilage from 5-month-old male mice. Femoral condyle (fc) and tibial plateau (tp) are shown. B) High magnification showing the femoral cartilage. The dotted line indicates the tidemark that separates the non-calcified (superficial) from the calcified zone. C, D) Hematoxylin and immunoperoxidase staining to detect aggrecan, collagen type II and FN in the femoral condyle (C) and tibial (D) articular cartilage. Scale bars, 50 μm.

To obtain *Fn1*^*RGE/-*^ mice expressing FN-RGE in cartilage, we intercrossed *Col2a-cre* transgenics with *Fn1*^*RGE/fl*^ compound mice (S1 Fig). The mutant mice were indistinguishable from *Col2a-cre;Fn1*^*wt/wt*^ controls (termed *Fn1*^*wt/wt*^) and displayed normal weight, body size and walking behaviour. After weaning (21 days), *Fn1*^*wt/wt*^ males weighed 9.3±1.3 g (n = 24) and *Fn1*^*RGE/-*^ males 8.7±1.7 g (n = 25). At the age of 4 months, the weight of *Fn1*^*wt/wt*^ increased to 30.7±2.3 g (n = 24) and of *Fn1*^*RGE/-*^ to 28.3±3.2 g (n = 25). To exclude defects in skeletal morphogenesis, we analysed the structure of the tibial growth plate in 3-month-old mice (S2 Fig). Chondrocyte number, the size of the growth plate and the columnar arrangement of cells were similar in *Fn1*^*wt/wt*^ and *Fn1*^*RGE/-*^ mice, which together with the normal body size suggests that growth of long bones proceeds normally in *Fn1*^*RGE/-*^ mice. Safranin-O staining of hind limb knee sections from 4.5–5 month-old *Fn1*^*RGE/-*^ male mice revealed normal cartilage surface and proteoglycan distribution in both, the femoral condyle and tibial plateau (Fig 1A and 1B). We measured the sizes of the non-calcified and calcified zones (separated by the tidemark, shown in Fig 1B) together with the spatial distribution of the ECM components in the femoral (Fig 1C) and tibial (Fig 1D) articular cartilages. The ratios of calcified/non-calcified areas in *Fn1*^*wt/wt*^ (1.95 ± 0.26) and in *Fn1*^*RGE/-*^ (1.91 ± 0.19) tibial condyles were similar. Immunostaining for aggrecan revealed a normal distribution around cells throughout the articular cartilage from *Fn1*^*RGE/-*^ and control mice. Type II collagen distribution was similar in control and *Fn1*^*RGE/-*^ mice. FN was expressed in the articular cartilage and in subchondral bone areas of control and *Fn1*^*RGE/-*^ mice.

To exclude a gene dosage effect we also analysed the articular cartilage of *Col2a1-Cre*;*Fn1*^*wt/fl*^ (*Fn1*^*wt/-*^) mice. Importantly, we did not find tissue abnormalities in the tibial growth plate (S2 Fig) or altered expression of ECM components (S3 Fig).

### Moderate mechanical load mildly affects articular cartilage of *Fn1*^*RGE/-*^ mice

**Fig 2:**
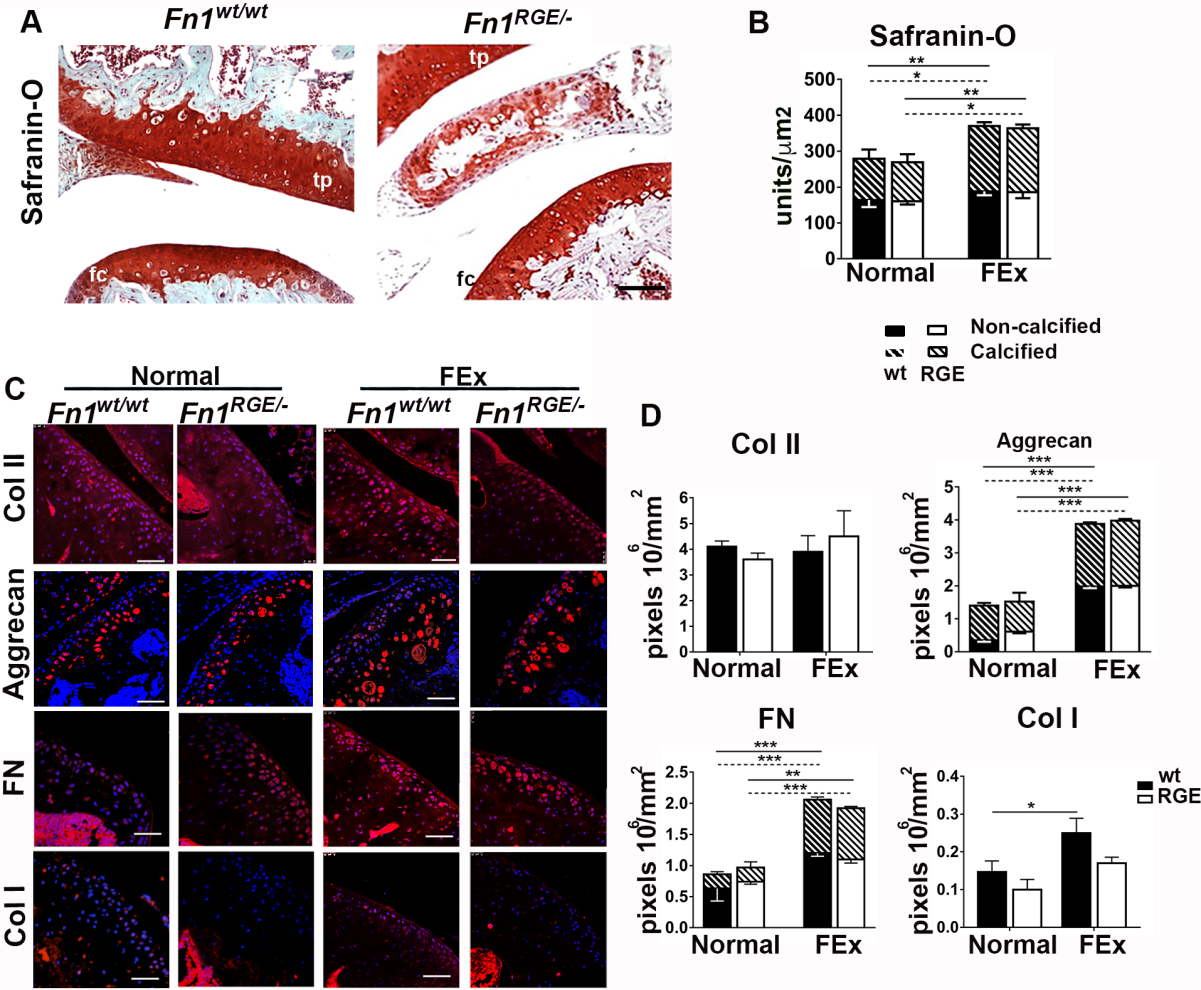
Influence of moderate mechanical load on the femoral knee joint cartilage. A) Representative Safranin-O stained femoral knee joint sections from *Fn1*^*wt/wt*^ and *Fn1*^*RGE/-*^ mice exposed to FEx. Femoral condyle (fc) and tibial plateau (tp) are shown. Scale bars, 50 μm. B) Quantification of Safranin-O densitometry in non-calcified and calcified zones of femoral cartilage from *Fn1*^*wt/wt*^ and *Fn1*^*RGE/-*^ mice with either normal activity (normal) or FEx. C) Immunofluorescence staining for type II collagen, aggrecan, FN and type I collagen in femoral knee cartilage from *Fn1*^*wt/wt*^ and *Fn1*^*RGE/-*^ mice with normal activity or FEx. Scale bars, 50 μm. D) Quantification of mean pixel density per mm^2^ in the articular cartilage. Aggrecan and FN were quantified in the non-calcified and calcified zones. Values represent mean ± SEM (n = 6 *Fn1*^*wt/wt*^ and 8 *Fn1*^*RGE/-*^ mice with FEx and n = 6 *Fn1*^*wt/wt*^ and 6 *Fn1*^*RGE/-*^ mice without FEx). The continuous line compares calcified zones and the dotted line compares non-calcified zones. Statistical significances: *p<0.05, **p<0.01 and ***p<0.001.

To further explore the response to moderate mechanical impact on the articular cartilage of *Fn1*^*RGE/-*^ males, we forced control and mutant mice to exercise (FEx) for 10 days (for details see Methods). Afterwards we analysed the morphology of the femoral and tibial knee articular cartilages by Safranin-O staining (Fig 2A), quantified its intensity in the femoral cartilage (Fig 2B) and determined the expression levels of major ECM components by immunofluorescence staining (Fig 2C). Densitometric quantification of Safranin-O staining in the two cartilage regions separated by the tidemark (shown in Fig 1B) revealed that FEx led to a similar increase of proteoglycan deposition in *Fn1*^*wt/wt*^ and *Fn1*^*RGE/-*^ mice (compare Fig 1B and Fig 2A). Interestingly, the proteoglycan increase was more evident in the calcified than in non-calcified zones (Fig 2B). Furthermore, *Fn1*^*wt/wt*^, *Fn1*^*wt/-*^ and *Fn1*^*RGE/-*^ mice displayed a normal distribution and similar numbers of chondrocytes in the articular cartilage (Fig 2A; S3 Fig). With respect to ECM components, FEx affected neither content nor distribution of collagen II in wild type and *Fn1*^*RGE/-*^ mice (Fig 2C and 2D) when compared to untrained animals (normal). Interestingly, aggrecan and FN levels significantly increased in the joint cartilage of *Fn1*^*wt/wt*^ and *Fn1*^*RGE/-*^ mice upon exercise (Fig 2C and 2D), however to a similar extent. FN and aggrecan levels increased in both the calcified and the non-calcified zones. The aggrecan deposition was slightly higher in the non-calcified than in the calcified zone (about 3-fold increase in the non-calcified versus 2-fold in calcified zones), whereas the increase of FN was higher in the calcified zone than in the non-calcified (about 1.5-fold in the non-calcified versus 3-fold in the calcified zone) and similarly in *Fn1*^*wt/wt*^ and *Fn1*^*RGE/-*^ mice. Interestingly, the level of type I collagen slightly increased in the articular cartilage from wild type mice and remained unchanged in *Fn1*^*RGE/-*^ mice (Fig 2C and 2D). Our results show that moderate mechanical loads induce a mild process of matrix remodelling in articular cartilage that proceeds independently of α5β1 integrin-mediated chondrocyte adhesion to FN.

### Partial meniscus resection combined with forced exercise accelerates OA in *Fn1*^*RGE/-*^ mice

**Fig 3:**
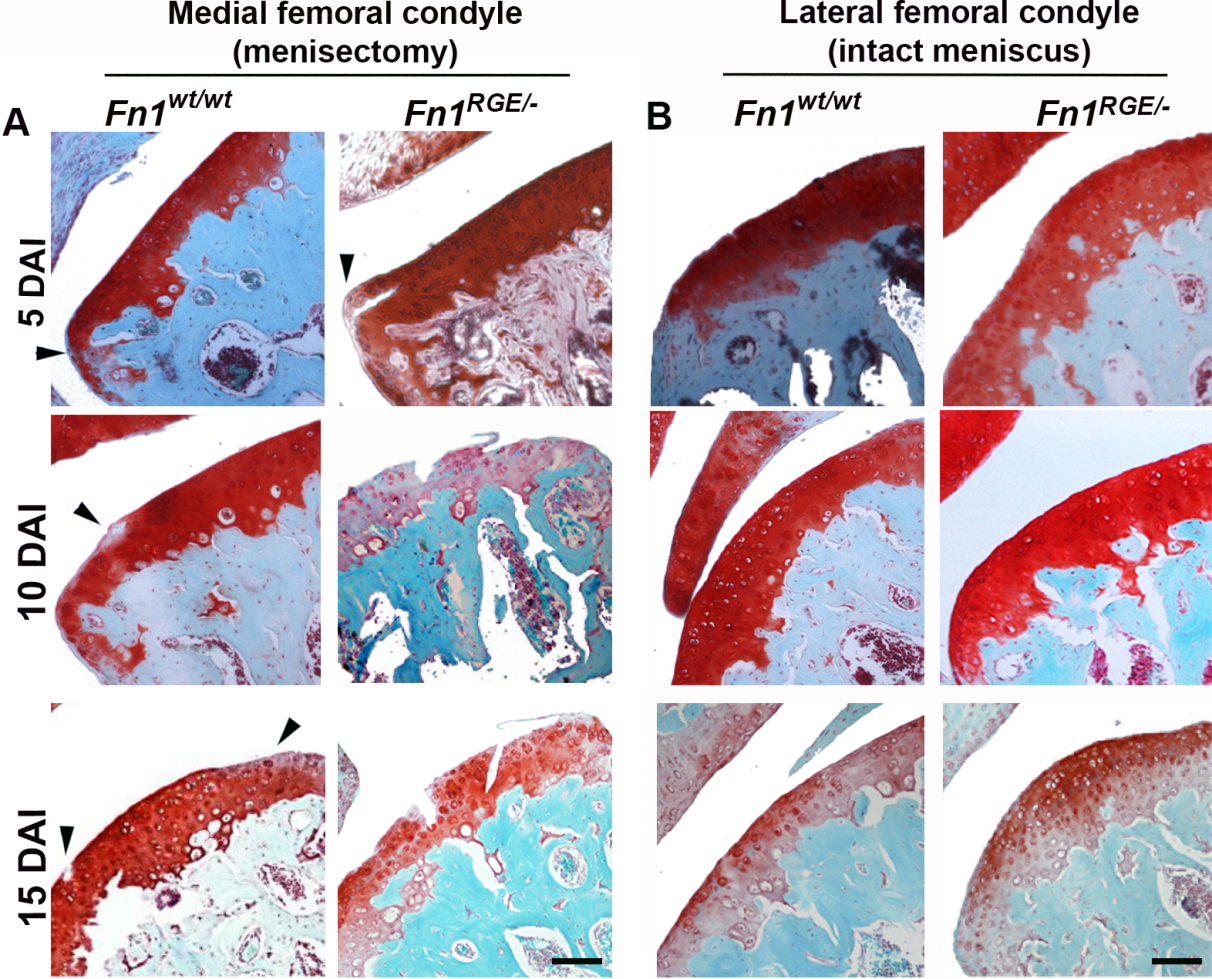
OA progression in *Fn1*^*wt/wt*^ and *Fn1*^*RGE/-*^ cartilage exposed to high mechanical load. A) Safranin-O staining of representative medial femoral knee cartilage sections from control and *Fn1*^*RGE/-*^ mice after exposure to high load at 5, 10 and 15 days after OA induction (DAI). B) Controls of the contralateral femoral condyles stained with Safranin-O. Scale bars, 50 μm.

To induce a high mechanical load, the medial meniscus in the right hind limb knee joint of wild type and *Fn1*^*RGE/-*^ littermates was partially resected and one day after surgery the mice were forced to perform daily exercise (for details see Methods). This model induces OA that starts in the femoral condyle and progresses similarly as in human OA [28]. The course of femoral cartilage destruction in the knee joint was visualised by Safranin-O staining 5, 10 and 15 days after induction (DAI) of menisectomy (Fig 3A; details in S4 Fig). We analysed 3 *Fn1*^*wt/wt*^ and 3 *Fn1*^*RGE/-*^ mice at 5 and 15 DAI, and 6 *Fn1*^*wt/wt*^ and 5 *Fn1*^*RGE/-*^ mice at 10 DAI. The signs of cartilage destruction were consistent between the mice of same genotype and OA stage. Importantly, none of the mice analysed showed signs of infection from surgery. Furthermore, we determined whether stress produced by the surgery affected tissue integrity by analysing the lateral femoral condyles of the operated knee (Fig 3B). In none of the mice analysed developed any sign of cartilage destruction. We also analysed 3 *Fn1*^*wt/-*^ mice at 10 DAI (S3C Fig) and found that the cartilage morphology was similar to that of *Fn1*^*wt/wt*^ mice of the same stage excluding that gene dosage affects the experimental outcome.

We applied a semi-quantitative scoring to estimate the OA severity following the OARSI recommendations [29]. In *Fn1*^*wt/wt*^ mice the morphological changes in the condylar cartilage were very mild at 5 DAI (Fig 3A). The articular cartilage layers are shown in S4 Fig. The first defect was visible at the margin of femoral condyle with slight proteoglycan loss in the SZ as determined by Safranin-O staining (arrowhead Fig 3A; S4 Fig). The middle zone of the condyle was normal. At 10 DAI, the condyle margins of *Fn1*^*wt/wt*^ as well as heterozygous *Fn1*^*wt/-*^ mice became fibrillated and the proteoglycans loss extended below the SZ. At 15 DAI, additional morphological changes developed such as cell rounding in the SZ, cell clustering and proteoglycan loss in all zones of the articular cartilage of *Fn1*^*wt/wt*^ mice. In *Fn1*^*RGE/-*^ mice, the articular cartilage showed severe signs of OA already at 5 DAI. The defects included cell clustering and irregular proteoglycan loss in the surface as well as in middle and deep zones of the articular cartilage. At 10 DAI the fibrillation was very pronounced and the proteoglycan loss affected the entire articular cartilage, on which also vertical breaches formed (Fig 3A). At 15 DAI the vertical clefts became highly abundant and extended deeply into the three zones of the articular cartilage. The severity of OA in the femoral condyles was scored according to OARSI recommendations (Table 1) and was higher in the medial condyles from *Fn1*^*RGE/-*^ than in *Fn1*^*wt/-*^ or *Fn1*^*wt/wt*^ mice. In the lateral control condyles the OARSI scores were 0, with the only exception of *Fn1*^*RGE/-*^ at 15 DAI that showed a slight proteoglycan decrease in some parts of the SZ.

**Table 1:** OARSI scoring of medial meniscus resected femoral condyles and lateral condyles from *Fn1*^*wt/wt*^ and *Fn1*^*RGE/-*^ mice exposed to high load.

Condyle	Genotype	OARSI histology scores		
		5 DAI	10 DAI	15 DAI
**Medial**	***Fn1***^***wt/wt***^	0.50 ± 0.10	1.41 ± 0.51	2.35 ± 0.12
	***Fn1***^***RGE/-***^	1.00 ± 0.15*	2.50 ± 0.25**	3.54 ± 0.51*
**Lateral**	***Fn1***^***wt/wt***^	0	0	0
	***Fn1***^***RGE/-***^	0	0	0.20 ± 0.05

Results represent mean ± SEM. Statistical significances:

*p<0.05 and

**p<0.01

**Fig 4:**
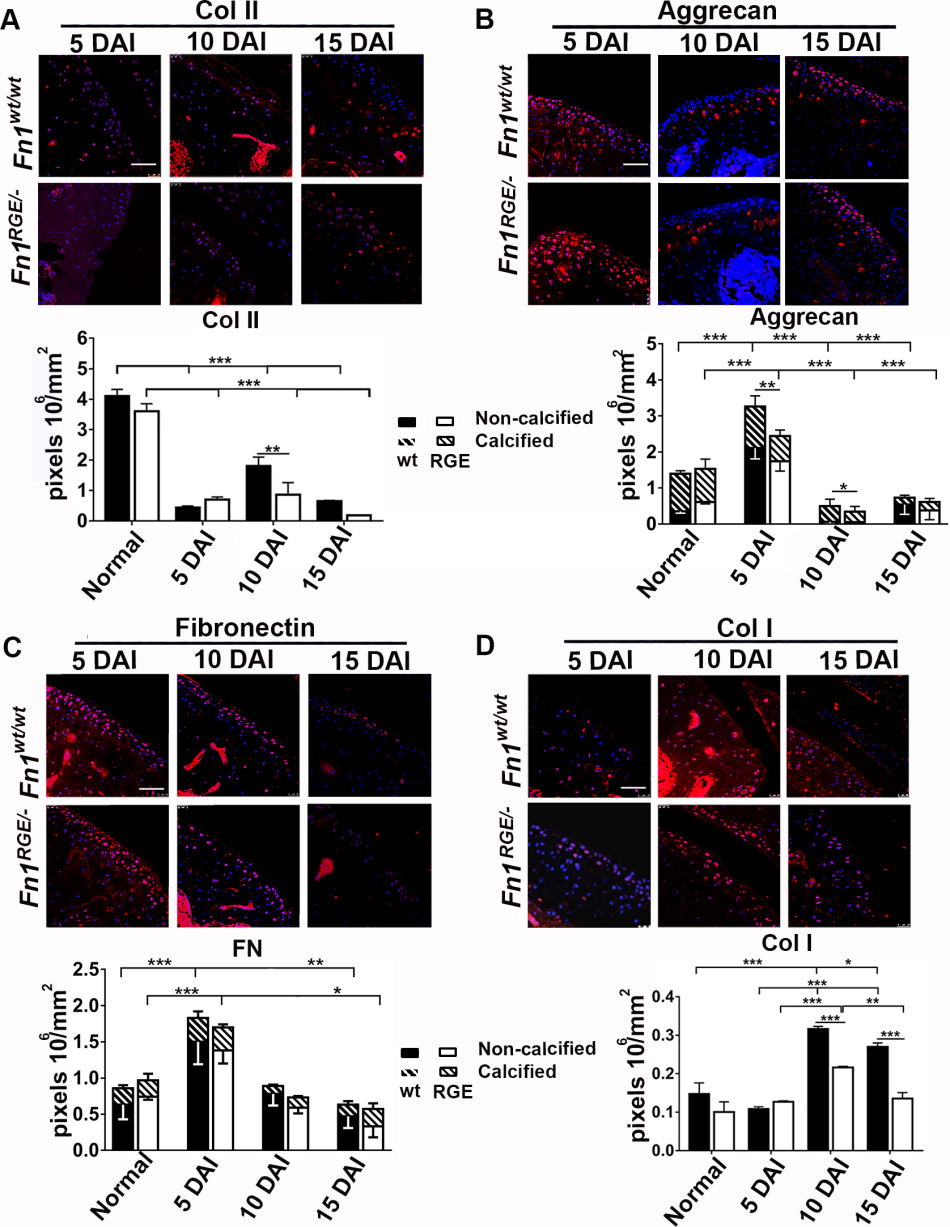
Immunofluorescence and quantification of ECM components in the femoral knee cartilage after exposure to high load. A) Immunofluorescence of collagen type II and quantification of pixel density per mm^2^ in the articular cartilage zone. Staining of cartilage from untrained (normal) mice is shown in Fig 2C. Aggrecan (B), FN (C) and collagen type I (D) staining and pixel density in articular cartilage. The pixel densities of aggrecan and FN were determined in the non-calcified and calcified zones. Results represent mean ± SEM. Scale bars, 50 μm. Statistical significances: *p<0.05, **p<0.01 and ***p<0.001.

Next, we performed immunostaining to determine the distribution and quantification of major ECM components of the articular cartilage including type II collagen (Fig 4A), aggrecan (Fig 4B), FN (Fig 4C) and type I collagen (Fig 4D). Collagen type II levels decreased rapidly in control and mutant articular cartilage after inducing OA, although more pronounced in *Fn1*^*RGE/-*^ than in *Fn1*^*wt/wt*^ mice (Fig 4A). Aggrecan and FN deposition were analysed in the non-calcified and calcified zones. Interestingly, at 5 DAI aggrecan levels increased by 2–fold in the *Fn1*^*wt/wt*^ articular cartilage and by 1.6-fold in *Fn1*^*RGE/-*^ mice and the increase was produced in the non-calcified zone. In the following days, aggrecan levels massively decreased, although significantly more pronounced in *Fn1*^*RGE/-*^ mice (Fig 4B). Significantly, the most dynamic changes in aggrecan levels were produced in the non-calcified zone, whereas in the calcified zone showed a progressive reduction in both, the *Fn1*^*wt/wt*^ and *Fn1*^*RGE/-*^ articular cartilage.

It has been reported that the cartilage ECM becomes degraded after injury and that chondrocytes respond with the production of a provisional matrix, whose major component is FN [2,30]. While FN significantly increased in 5 DAI articular cartilage of both *Fn1*^*RGE/-*^ as well as *Fn1*^*wt/wt*^ mice (Fig 4C), the FN content significantly and similarly decreased in wild type and *Fn1*^*RGE/-*^ mice at 10 and 15 DAI. As with aggrecan, major changes of FN content were produced in the non-calcified zones. In agreement with previous reports [31], after injury the articular cartilage remodelling is initiated in the non-calcified, upper cartilage. The collagen I content was unaltered at 5 DAI and increased 2-fold at 10 and 15 DAI in condyles of *Fn1*^*wt/wt*^ mice. In contrast, collagen I levels only slightly rose in *Fn1*^*RGE/-*^ mice at 10 DAI indicating that FN-RGE expressing cartilage is less efficient in assembling a collagen type I–containing provisional ECM.

### Increased expression of MMPs in stressed *Fn1*^*RGE/-*^ articular cartilage

**Fig 5:**
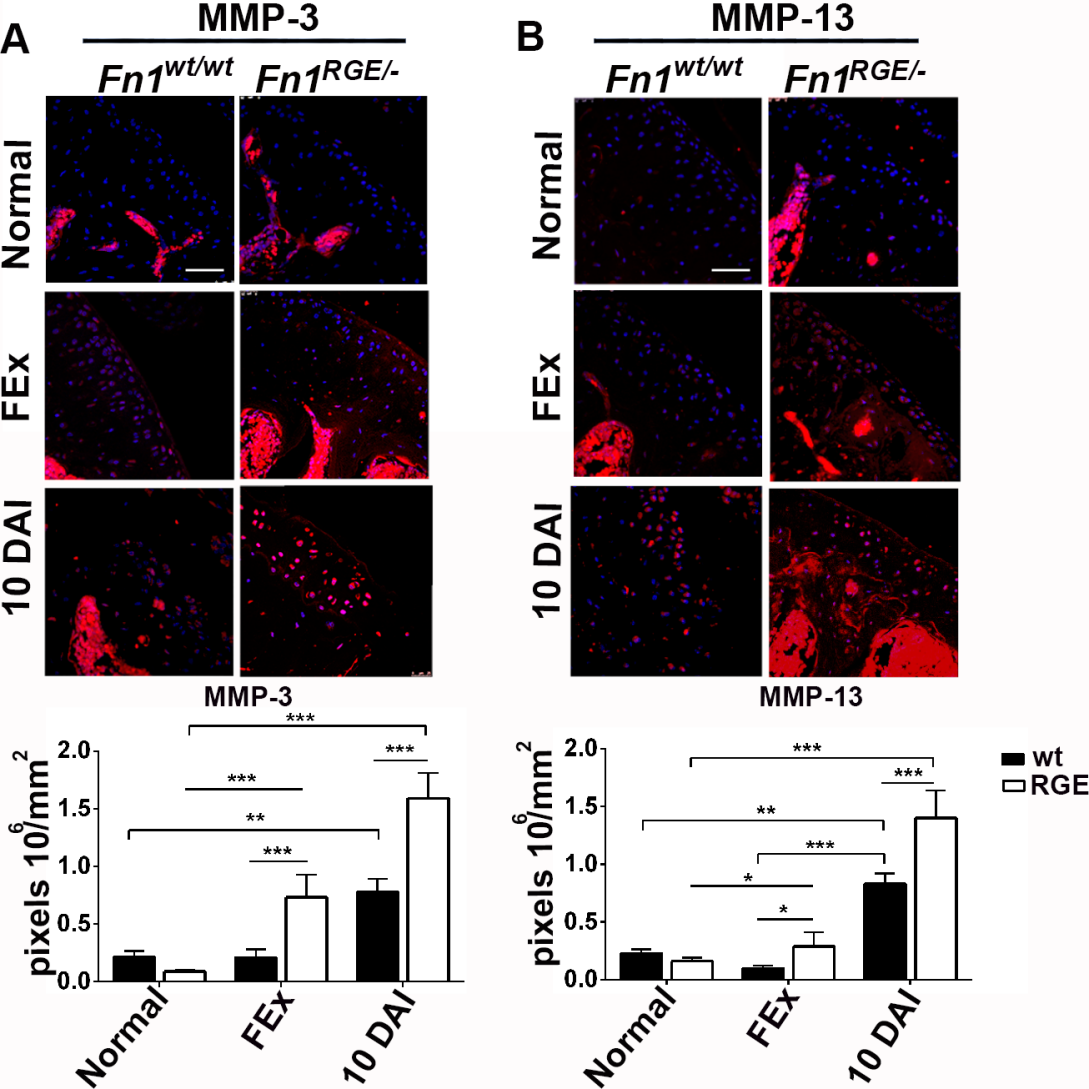
Immunofluorescence and quantification of MMP-3 and MMP-13 in femoral knee cartilage exposed to high load. A) Immunofluorescence staining for MMP-3 levels and quantification of pixel density per mm^2^ in the articular cartilage of mice 10 days after exposure to normal activity (normal), moderate load (FEx) and high load (10 DAI). B) MMP-13 levels. Values represent mean ± SEM. Scale bars, 50 μm. Statistical significances: *p<0.05, **p<0.01 and ***p<0.001.

Remodelling of the articular cartilage ECM after injury is accompanied by an increased expression and/or activity of MMPs, which degrade deteriorated ECM and thereby allow production of a new functional matrix [2]. We focused our analysis on MMP-3 and MMP-13 (Fig 5), whose expression plays a major role in cartilage matrix degradation during OA and was shown to be stimulated by integrin bound FN fragments [4,14,17]. The moderate mechanical load produced by forced exercise did not influence MMP-13 and MMP-3 levels in *Fn1*^*wt/wt*^ mice, whereas MMP-3 expression increased 3-fold in articular cartilage derived from *Fn1*^*RGE/-*^ mice (Fig 5A). Exposure to forced exercise together with partial meniscus resection increased MMP-3 and MMP-13 levels significantly at 10 DAI both in *Fn1*^*RGE/-*^ and *Fn1*^*wt/wt*^ mice, although the increase was more pronounced in *Fn1*^*RGE/-*^ cartilage. Specifically, MMP-3 increased 3-fold in *Fn1*^*wt/wt*^ and 7.8-fold in *Fn1*^*RGE/-*^, and MMP-13 increased 4.5-fold in *Fn1*^*wt/wt*^ and 6.4-fold in *Fn1*^*RGE/-*^ cartilage. These data indicate that despite the inability of FN fragments generated from FN-RGE to bind to and signal via α5β1 integrins, the MMP-3 and MMP-13 increase after cartilage injury is higher in *Fn1*^*RGE/-*^ than in control articular cartilage.

## Discussion

Our study reveals that experimental OA induced by menisectomy followed by forced exercise accelerates cartilage degradation in mice that lack α5β1-mediated adhesion of chondrocytes to FN.

There are two integrin classes that bind FN via the RGD motif, which results in cell adhesion and signalling: α5β1 and αv-class integrins [32]. The binding to both integrin classes controls the assembly of the fibrillar FN network, which is, in turn, believed to serve as scaffold for collagen and proteoglycans organization. The role of FN and FN receptors for cartilage repair is not well understood. On one hand, FN has been suggested to play a structural or instructive role in the cartilage [13], which is changed and adapted by mechanical stress that in turn promotes further FN synthesis, induces chondrocyte proliferation [33,34] and prevents death of chondrocytes via α5β1 integrin signalling [12]. In line with these findings, inhibition of α5β1 integrin with RGD peptides or anti-α5β1 antibodies [17] or treatments with RGD-containing FN fragments [35,36], but not with the native FN molecule [37], were shown to accelerate cartilage degradation. On the other hand, however, it was also shown that absent α5 integrin signalling by inhibiting α5 integrin expression arrests cartilage destruction induced by FN fragments in explant experiments [16]. This view gained further support by a recent report by Candela et al. [38], which demonstrated that conditional deletion of the α5 integrin gene (*Itga5*) in synovial joints results in reduced matrix loss and reduced OA.

To directly test whether the expression of α5β1 integrins on chondrocytes unable to bind FN has *in vivo* consequence for cartilage ECM remodelling or function after exposure to mechanical loads, we expressed FN-RGE in articular cartilage of mice. FN-RGE was shown by us and others to bind αv-class integrins but not α5β1 integrins [24,25]. Furthermore, FN-RGE is assembled into a fibrillar FN matrix in tissues of mice, although the fibrils appeared slightly thicker and shorter [24,25]. The consequence of the inability of FN-RGE to bind α5β1 is embryonic lethality at about E9.5 characterized by vascular defects and a truncated body axis due to severe defects of somitogénesis, indicating that FN-RGE matrix signalling is not functional in certain mesoderm-derived tissues [24,25]. In our current study, we found that meniscus resection in *Fn1*^*RGE/-*^ mice followed by forced exercise accelerated the onset as well as course of OA. Their OA cartilage was characterized by increased loss of proteoglycans and collagen II, lack of collagen type I upregulation and increased expression of MMP-13 and MMP-3. The increased MMP-13 and -3 expression provides a reasonable explanation for the accelerated reduction of collagen type II and proteoglycans. The altered biochemical features impair the mechanical stability of the *Fn1*^*RGE/-*^ articular cartilage, similarly as observed upon application of compounds disrupting the FN-integrin interaction such as soluble FN fragments containing the RGD motif in the FNIII10 region or inhibitory antibodies [14–17]. It is conceivable that unligated α5β1 integrins on the surface of articular chondrocytes cannot establish a productive link to actomyosin-mediated forces, which results in the activation of caspases and induction of apoptosis as well as the induction of ECM metalloproteinases, as was described for unligated α5β1 integrins in tumour cells [39,40]. Interestingly, although the articular cartilage of *Fn1*^*RGE/-*^ mice exposed to forced exercise without menisectomy did not show signs of proteoglycan loss or cartilage destruction, our careful analysis revealed that some small changes were evident. For example, MMP-3 and MMP-13 levels were slightly but significantly increased in FN-RGE articular cartilage. Moreover, exercise induced upregulation of collagen type I in *Fn1*^*wt/wt*^ mice but not in *Fn1*^*RGE/-*^. The upregulation of collagen I and FN is an important part of a matrix remodelling process occurring in the articular cartilage and results in the formation of a provisional matrix which is essential for tissue regeneration [41]. Although collagen I can spontaneously form fibrils *in vitro*, it has been demonstrated that an integrin-assembled matrix of FN is necessary *in vivo* to orientate and organize collagen I fibrils [42–44]. Possibly a structurally altered FN-RGE matrix will fail to properly assemble collagen I fibrils and thus will make them accessible to MMP-mediated degradation, which in turn contributes to impaired articular cartilage regeneration.

It is interesting, however, that the lack of FN-α5β1 adhesion by chondrocytes *per se* did not impair cartilage development and homeostasis of non-stressed mice. Cartilage morphology as well as content and distribution of the major ECM components was similar in the articular knee joint cartilage of *Fn1*^*RGE/-*^ and *Fn1*^*wt/wt*^ mice. Despite its faithful expression, FN-RGE interfered neither with cartilage development nor with a normal morphology of articular cartilage. A possible explanation is that collagen type II is nucleated and organized differently to collagen I fibrils. Indeed, it has been demonstrated that collagen II assembles a normal network in a FN-independent manner *in vivo* [44]. Furthermore, during cartilage development the expression of FN and collagen I precedes the expression of collagen II and aggrecan that will eventually form the definitive cartilage matrix [41]. The *Fn1*^*RGE/-*^ genotype is conditionally regulated by the collagen II promotor and thus we assume that in the initial matrix the FN was wild type.

Taken together, our results support that intense mechanical load triggers a spatiotemporal regulation of ECM synthesis and degradation in the articular cartilage, which is controlled by the interaction of FN with α5β1 integrins. If the FN fibrillar network is missing or assembled by fibrils that cannot bind and activate α5β1 integrins, articular cartilage chondrocytes increase MMPs expression, which in turn jeopardizes tissue regeneration leading to OA. Our results underscore the critical role of FN-α5β1 adhesion as a mechanical sensor of ECM not only during tumour invasion [40,45] but also during tissue regeneration.

## Supporting information

S1 Fig Wild type and mutant *Fn1* genes. A) The *Fn1* wild type (*Fn1*^wt^), mutant (*Fn1*^RGE^), floxed (*Fn1*^fl^) and null (*Fn1*^-^) alleles and location (arrows) of primers used for PCR genotyping. B) *Fn1* alleles in *Col2a1-Cre;Fn1* ^*RGE/fl*^ mice, which express only the *Fn1*^RGE^ in cartilage, whereas *Fn1*^fl^ allele allows normal FN expression in all other tissues. C) PCR genotyping with primers located in intron 30. D) PCR genotyping for Cre-mediated recombination of the floxed allele in cartilage from *Col2a1-Cre;Fn1*^*RGE/fl*^ mice using the primers located in exon 1 and intron 1. The mice were genotyped with genomic DNA isolated from tail (T) and cartilage (C) tissues.
(TIF)

S2 Fig Growth plate morphology in the tibia of 3-month-old mice. Hematoxylin-Chromotrope-2R staining indicates the growth plate structure. The size and the typical columnar arrangement of the chondrocytes were similar in *Fn1*^*wt/wt*^, *Fn1*^*wt/-*^ and *Fn1*^*RGE/-*^ mice. Scale bar, 100 μm in the low magnification and 50 μm in the detail of growth plate.
(TIF)

S3 Fig Articular cartilage of the femoral condyle from *Fn1*^*wt/-*^ mice. A) Morphology and ECM proteins expression in articular cartilage from *Fn1*^*wt/-*^ femoral condyles (n = 3). B) Safranin-O staining after forced exercise (FEx) during 10 days (n = 3). C) Safranin-O staining 10 days after exposure to high load (10 DAI) (n = 3). Scale bars, 50 μm.
(TIF)

S4 Fig Articular cartilage of *Fn1*^*wt/wt*^ and *Fn1*^*RGE/-*^ mice. Safranin-O staining of sections of the femoral knee cartilage from control and *Fn1*^*RGE/-*^ littermates after exposure to high load. High magnifications are depicted. Scale bar, 50 μm.
(TIF)

## References

[1] Loeser RF. Molecular mechanisms of cartilage destruction in osteoarthritis. J Musculoskelet Neuronal Interact. 2008;8: 303–306. 19147949

[2] Stoffels JMJ, Zhao C, Baron W. Fibronectin in tissue regeneration: timely disassembly of the scaffold is necessary to complete the build. Cell Mol Life Sci. 2013;70: 4243–4253. doi: 10.1007/s00018-013-1350-0 23756580 10.1007/s00018-013-1350-0 PMC11113129

[3] Homandberg GA, Guo D, Ray LM, Ding L. Mixtures of glucosamine and chondroitin sulfate reverse fibronectin fragment mediated damage to cartilage more effectively than either agent alone. Osteoarthritis and Cartilage. 2006;14: 793–806. doi: 10.1016/j.joca.2006.02.003 16581272 10.1016/j.joca.2006.02.003

[4] Stanton H, Ung L, Fosang AJ. The 45 kDa collagen-binding fragment of fibronectin induces matrix metalloproteinase-13 synthesis by chondrocytes and aggrecan degradation by aggrecanases. Biochem J. Portland Press Ltd; 2002;364: 181–190. 11988091 10.1042/bj3640181 PMC1222560

[5] Sofat N. Analysing the role of endogenous matrix molecules in the development of osteoarthritis. International Journal of Experimental Pathology. 2009;90: 463–479. doi: 10.1111/j.1365-2613.2009.00676.x 19765101 10.1111/j.1365-2613.2009.00676.x PMC2768145

[6] Yasuda T. Cartilage destruction by matrix degradation products. Mod Rheumatol. 2006;16: 197–205. doi: 10.1007/s10165-006-0490-6 16906368 10.1007/s10165-006-0490-6 PMC2780665

[7] Yasuda T, Poole AR. A fibronectin fragment induces type II collagen degradation by collagenase through an interleukin-1-mediated pathway. Arthritis & Rheumatism. John Wiley & Sons, Inc; 2002;46: 138–148. doi: 10.1002/1529-0131(200201)46:1<138::AID-ART10051>3.0.CO;2-K 11817586 10.1002/1529-0131(200201)46:1<138::AID-ART10051>3.0.CO;2-K

[8] Leiss M, Beckmann K, Girós A, Costell M, Fässler R. The role of integrin binding sites in fibronectin matrix assembly in vivo. Curr Opin Cell Biol. 2008;20: 502–507. doi: 10.1016/j.ceb.2008.06.001 18586094 10.1016/j.ceb.2008.06.001

[9] Loeser RF. Integrins and chondrocyte-matrix interactions in articular cartilage. Matrix Biol. Elsevier B.V; 2014;39: 11–16. doi: 10.1016/j.matbio.2014.08.007 25169886 10.1016/j.matbio.2014.08.007 PMC4699681

[10] Tian J, Zhang F-J, Lei G-H. Role of integrins and their ligands in osteoarthritic cartilage. Rheumatol Int. 2014;35: 787–798. doi: 10.1007/s00296-014-3137-5 25261047 10.1007/s00296-014-3137-5

[11] Aszódi A. 1 integrins regulate chondrocyte rotation, G1 progression, and cytokinesis. Genes Dev. 2003;17: 2465–2479. doi: 10.1101/gad.277003 14522949 10.1101/gad.277003 PMC218082

[12] Pulai JI, Del Carlo M, Loeser RF. The ?5?1 integrin provides matrix survival signals for normal and osteoarthritic human articular chondrocytes in vitro. Arthritis & Rheumatism. 2002;46: 1528–1535. doi: 10.1002/art.10334 12115183 10.1002/art.10334

[13] Singh P, Schwarzbauer JE. Fibronectin and stem cell differentiation—lessons from chondrogenesis. Journal of Cell Science. 2012;125: 3703–3712. doi: 10.1242/jcs.095786 22976308 10.1242/jcs.095786 PMC3462078

[14] Del Carlo M, Schwartz D, Erickson EA, Loeser RF. Endogenous production of reactive oxygen species is required for stimulation of human articular chondrocyte matrix metalloproteinase production by fibronectin fragments. Free Radic Biol Med. 2007;42: 1350–1358. doi: 10.1016/j.freeradbiomed.2007.01.035 17395008 10.1016/j.freeradbiomed.2007.01.035 PMC1892212

[15] Werb Z, Tremble PM, Behrendtsen O, Crowley E, Damsky CH. Signal transduction through the fibronectin receptor induces collagenase and stromelysin gene expression. J Cell Biol. The Rockefeller University Press; 1989;109: 877–889. 2547805 10.1083/jcb.109.2.877 PMC2115739

[16] Homandberg G. Antisense oligonucleotides to the integrin receptor subunit alpha5 decrease fibronectin fragment mediated cartilage chondrolysis. Osteoarthritis and Cartilage. 2002;10: 381–393. doi: 10.1053/joca.2002.0524 12027539 10.1053/joca.2002.0524

[17] Forsyth CB, Pulai J, Loeser RF. Fibronectin fragments and blocking antibodies to alpha2beta1 and alpha5beta1 integrins stimulate mitogen-activated protein kinase signaling and increase collagenase 3 (matrix metalloproteinase 13) production by human articular chondrocytes. Arthritis & Rheumatism. Wiley Subscription Services, Inc., A Wiley Company; 2002;46: 2368–2376. doi: 10.1002/art.10502 12355484 10.1002/art.10502

[18] Kurtis MS, Schmidt TA, Bugbee WD, Loeser RF, Sah RL. Integrin-mediated adhesion of human articular chondrocytes to cartilage. Arthritis & Rheumatism. 2003;48: 110–118. doi: 10.1002/art.10704 12528111 10.1002/art.10704

[19] Almonte-Becerril M, Costell M, Kouri JB. Changes in the integrins expression are related with the osteoarthritis severity in an experimental animal model in rats. J Orthop Res. 2014;32: 1161–1166. doi: 10.1002/jor.22649 24839051 10.1002/jor.22649

[20] Singh A, Rajasekaran N, Hartenstein B, Szabowski S, Gajda M, Angel P, et al Collagenase-3 (MMP-13) deficiency protects C57BL/6 mice from antibody-induced arthritis. Arthritis Research & Therapy. 2013;15: R222 doi: 10.1186/ar4423 24369907 10.1186/ar4423 PMC3979078

[21] Jüngel A, Ospelt C, Lesch M, Thiel M, Sunyer T, Schorr O, et al Effect of the oral application of a highly selective MMP-13 inhibitor in three different animal models of rheumatoid arthritis. Ann Rheum Dis. BMJ Publishing Group Ltd; 2010;69: 898–902. doi: 10.1136/ard.2008.106021 19497915 10.1136/ard.2008.106021 PMC2925150

[22] Little CB, Barai A, Burkhardt D, Smith SM, Fosang AJ, Werb Z, et al Matrix metalloproteinase 13-deficient mice are resistant to osteoarthritic cartilage erosion but not chondrocyte hypertrophy or osteophyte development. Arthritis & Rheumatism. Wiley Subscription Services, Inc., A Wiley Company; 2009;60: 3723–3733. doi: 10.1002/art.25002 19950295 10.1002/art.25002 PMC2832925

[23] Wang M, Sampson ER, Jin H, Li J, Ke QH, Im H-J, et al MMP13 is a critical target gene during the progression of osteoarthritis. Arthritis Research & Therapy. BioMed Central; 2013;15: R5 doi: 10.1186/ar4133 23298463 10.1186/ar4133 PMC3672752

[24] Girós A, Grgur K, Gossler A, Costell M. α5β1 integrin-mediated adhesion to fibronectin is required for axis elongation and somitogenesis in mice. PLoS ONE. 2011;6: e22002 doi: 10.1371/journal.pone.0022002 21799763 10.1371/journal.pone.0022002 PMC3142108

[25] Takahashi S, Leiss M, Moser M, Ohashi T, Kitao T, Heckmann D, et al The RGD motif in fibronectin is essential for development but dispensable for fibril assembly. J Cell Biol. 2007;178: 167–178. doi: 10.1083/jcb.200703021 17591922 10.1083/jcb.200703021 PMC2064432

[26] Sakai T, Johnson KJ, Murozono M, Sakai K, Magnuson MA, Wieloch T, et al Plasma fibronectin supports neuronal survival and reduces brain injury following transient focal cerebral ischemia but is not essential for skin-wound healing and hemostasis. Nat Med. 2001;7: 324–330. doi: 10.1038/85471 11231631 10.1038/85471

[27] Sakai K, Hiripi L, Glumoff V, Brandau O, Eerola R, Vuorio E, et al Stage-and tissue-specific expression of a Col2a1-Cre fusion gene in transgenic mice. Matrix Biol. 2001;19: 761–767. 11223335 10.1016/s0945-053x(00)00122-0

[28] Almonte-Becerril M, Navarro-Garcia F, Gonzalez-Robles A, Vega-Lopez MA, Lavalle C, Kouri JB. Cell death of chondrocytes is a combination between apoptosis and autophagy during the pathogenesis of Osteoarthritis within an experimental model. Apoptosis. 2010;15: 631–638. doi: 10.1007/s10495-010-0458-z 20091349 10.1007/s10495-010-0458-z

[29] Glasson SS, Chambers MG, Van Den Berg WB, Little CB. The OARSI histopathology initiative—recommendations for histological assessments of osteoarthritis in the mouse. Osteoarthritis and Cartilage. Elsevier Ltd; 2010;18: S17–S23. doi: 10.1016/j.joca.2010.05.025 20864019 10.1016/j.joca.2010.05.025

[30] Brown RA, Jones KL. The synthesis and accumulation of fibronectin by human articular cartilage. J Rheumatol. 1990;17: 65–72. 2313677

[31] Pfander D, Rahmanzadeh R, Scheller EE. Presence and distribution of collagen II, collagen I, fibronectin, and tenascin in rabbit normal and osteoarthritic cartilage. J Rheumatol. 1999;26: 386–394. 9972974

[32] Schiller HB, Hermann M-R, Polleux J, Vignaud T, Zanivan S, Friedel CC, et al beta1- and alphav-class integrins cooperate to regulate myosin II during rigidity sensing of fibronectin-based microenvironments. Nat Cell Biol. Nature Publishing Group; 2013;15: 625–636. doi: 10.1038/ncb2747 23708002 10.1038/ncb2747

[33] Perera PM, Wypasek E, Madhavan S, Rath-Deschner B, Liu J, Nam J, et al Mechanical signals control SOX-9, VEGF, and c-Myc expression and cell proliferation during inflammation via integrin-linked kinase, B-Raf, and ERK1/2-dependent signaling in articular chondrocytes. Arthritis Research & Therapy. BioMed Central; 2010;12: R106 doi: 10.1186/ar3039 20509944 10.1186/ar3039 PMC2911896

[34] Wright MO, Nishida K, Bavington C, Godolphin JL, Dunne E, Walmsley S, et al Hyperpolarisation of cultured human chondrocytes following cyclical pressure-induced strain: evidence of a role for alpha 5 beta 1 integrin as a chondrocyte mechanoreceptor. J Orthop Res. Wiley Subscription Services, Inc., A Wiley Company; 1997;15: 742–747. doi: 10.1002/jor.1100150517 9420605 10.1002/jor.1100150517

[35] Yasuda T, Poole AR. A fibronectin fragment induces type II collagen degradation by collagenase through an interleukin-1-mediated pathway. Arthritis & Rheumatism. John Wiley & Sons, Inc; 2002;46: 138–148. doi: 10.1002/1529-0131(200201)46:1<138::AID-ART10051>3.0.CO;2-K 11817586 10.1002/1529-0131(200201)46:1<138::AID-ART10051>3.0.CO;2-K

[36] Yasuda T. Cartilage destruction by matrix degradation products. Mod Rheumatol. 2006;16: 197–205. doi: 10.1007/s10165-006-0490-6 16906368 10.1007/s10165-006-0490-6 PMC2780665

[37] Signal transduction through the fibronectin receptor induces collagenase and stromelysin gene expression. The Rockefeller University Press; 1989;109: 877–889. 2547805 10.1083/jcb.109.2.877 PMC2115739

[38] Candela ME, Wang C, Gunawardena AT, Zhang K, Cantley L, Yasuhara R, et al Alpha 5 Integrin Mediates Osteoarthritic Changes in Mouse Knee Joints. Williams BO, editor. PLoS ONE. Public Library of Science; 2016;11: e0156783–14. doi: 10.1371/journal.pone.0156783 27280771 10.1371/journal.pone.0156783 PMC4900574

[39] Stupack DG, Puente XS, Boutsaboualoy S, Storgard CM, Cheresh DA. Apoptosis of adherent cells by recruitment of caspase-8 to unligated integrins. J Cell Biol. 2001;155: 459–470. doi: 10.1083/jcb.200106070 11684710 10.1083/jcb.200106070 PMC2150834

[40] Varner JA, Emerson DA, Juliano RL. Integrin alpha 5 beta 1 expression negatively regulates cell growth: reversal by attachment to fibronectin. Mol Biol Cell. American Society for Cell Biology; 1995;6: 725–740. 7579691 10.1091/mbc.6.6.725 PMC301232

[41] Singh P, Schwarzbauer JE. Fibronectin and stem cell differentiation—lessons from chondrogenesis. Journal of Cell Science. 2012;125: 3703–3712. doi: 10.1242/jcs.095786 22976308 10.1242/jcs.095786 PMC3462078

[42] McDonald JA. Role of fibronectin in collagen deposition: Fab' to the gelatin-binding domain of fibronectin inhibits both fibronectin and collagen organization in fibroblast extracellular matrix. J Cell Biol. 1982;92: 485–492. doi: 10.1083/jcb.92.2.485 7061591 10.1083/jcb.92.2.485 PMC2112086

[43] Li S, Van Den Diepstraten C, D'Souza SJ, Chan BMC, Pickering JG. Vascular smooth muscle cells orchestrate the assembly of type I collagen via alpha2beta1 integrin, RhoA, and fibronectin polymerization. AJPA. American Society for Investigative Pathology; 2003;163: 1045–1056. 10.1016/s0002-9440(10)63464-5 PMC1868248 12937145

[44] Kadler KE, Hill A, Canty-Laird EG. Collagen fibrillogenesis: fibronectin, integrins, and minor collagens as organizers and nucleators. Curr Opin Cell Biol. 2008;20: 495–501. doi: 10.1016/j.ceb.2008.06.008 18640274 10.1016/j.ceb.2008.06.008 PMC2577133

[45] Stupack DG. Get a ligand, get a life: integrins, signaling and cell survival. Journal of Cell Science. 2002;115: 3729–3738. doi: 10.1242/jcs.00071 12235283 10.1242/jcs.00071

