# Supplementary figures and images for: Genetic abrogation of the fibronectin-α5β1 integrin interaction in articular cartilage aggravates osteoarthritis in mice
Source: PLoS One. 2018 Jun 5;13(6):e0198559. doi: 10.1371/journal.pone.0198559 (PMC5988303; doi:10.1371/journal.pone.0198559)

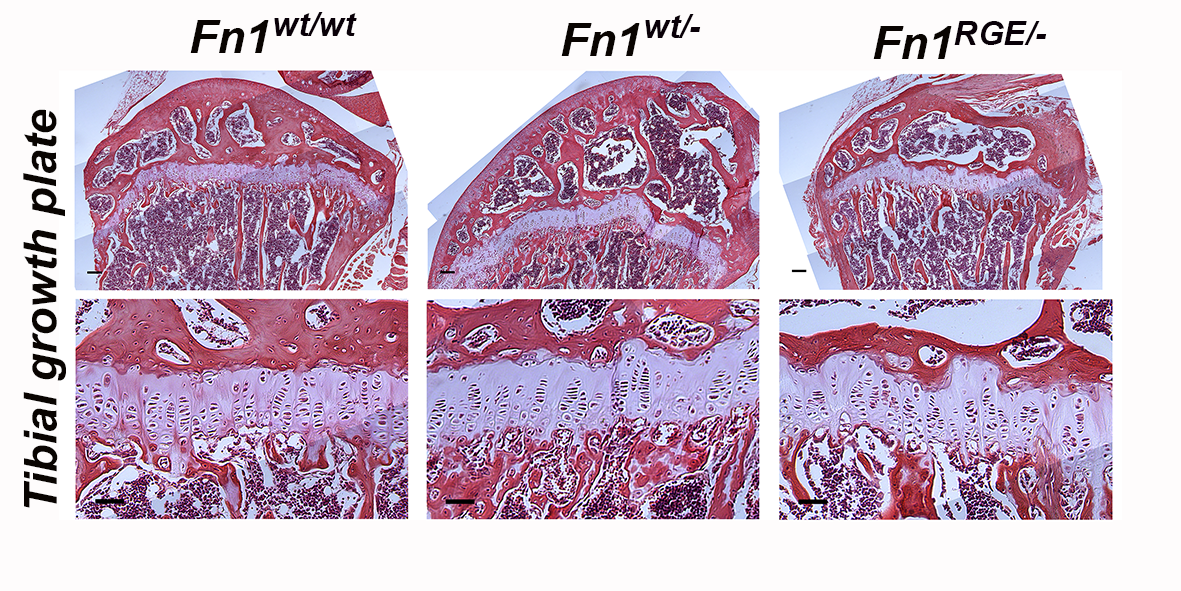

Supplement: S2 Fig — Hematoxylin-Chromotrope-2R staining indicates the growth plate structure. The size and the typical columnar arrangement of the chondrocytes were similar in Fn1wt/wt, Fn1wt/- and Fn1RGE/- mice. Scale bar, 100 μm in the low magnification and 50 μm in the detail of growth plate. (TIF) [file pone.0198559.s002.tif]
